# Design, Materials, and Mechanobiology of Biodegradable Scaffolds for Bone Tissue Engineering

**DOI:** 10.1155/2015/729076

**Published:** 2015-03-26

**Authors:** Marco A. Velasco, Carlos A. Narváez-Tovar, Diego A. Garzón-Alvarado

**Affiliations:** ^1^Studies and Applications in Mechanical Engineering Research Group (GEAMEC), Universidad Santo Tomás, Bogotá, Colombia; ^2^Biomimetics Laboratory and Numerical Methods and Modeling Research Group (GNUM), Instituto de Biotecnología (IBUN), Universidad Nacional de Colombia, Bogotá, Colombia

## Abstract

A review about design, manufacture, and mechanobiology of biodegradable scaffolds for bone tissue engineering is given. First, fundamental aspects about bone tissue engineering and considerations related to scaffold design are established. Second, issues related to scaffold biomaterials and manufacturing processes are discussed. Finally, mechanobiology of bone tissue and computational models developed for simulating how bone healing occurs inside a scaffold are described.

## 1. Introduction

Bones are rigid organs that consist of osseous tissue, bone marrow, endosteum, periosteum, cartilage, nerves, and vascular channels constituting the skeleton of vertebrate animals. Osseous tissue, which fulfills mechanical functions, is formed by connective tissue cells such as osteocytes, osteoblasts, and osteoclasts [1, 2] in an extracellular matrix composed mainly of minerals, proteins, and water. The bone composition and configuration will vary according to factors such as the anatomical location, supported load, age and gender of the individual, and the possible diseases that he or she could suffer [3, 4]. In regard to bone composition, mineral phase is between 60 and 70 wt.% and water between 5 and 10 wt.%, while the remaining portion is an organic matrix of collagen and other proteins.

The mineral phase of bone is essentially a calcium phosphate, called hydroxyapatite, presented in the form of nanocrystals with sizes between 25 and 50 nm in length [5]. Variations in the chemical composition of hydroxyapatite modify its physical properties, specially its solubility [6]. On the other hand, its biochemical properties mainly depend on the organic phase of the extracellular matrix of bone. Approximately 90% of the organic phase is formed by type I collagen. The remainder consists of proteins, lipids, and other macromolecules such as growth factors, cytokines, osteonectin, osteopontin, osteocalcin, osteoinductive proteins, sialoproteins, proteoglycans, phosphoproteins, and phospholipids [7–9]. Mineral and organic phases determine the mechanical properties of bone as a composite.

According to its structure, osseous tissue may be cancellous (trabecular) or cortical (lamellar). Cancellous bone is a network of interconnected porosities, ranging between 50% to 90% of void space, with a solid portion which is formed of struts and plates that can adopt different configurations. It is located at the epiphysis of long bones and the interior of cuboid bones. Cortical tissue is located at the bone surface and it has a homogeneous and compact macrostructure. It is found mainly at the bone diaphysis and its thickness varies according to the bone anatomical location. Cortical bone consists of structural and functional units called osteons or Haversian systems. Osteons are arranged along the bone. Inside the osteons, there are small spaces or lacunae where osteocytes are housed. Osteons contains tiny channels or canaliculi to provide nutrient and oxygen to the cells. Along the center of the osteons there is a central channel with vessels and nerves.

Bones have mechanical, synthetic, and metabolic functions. The mechanical functions are protection of internal organs, body support, and interaction with muscles and tendons to generate body movement [5]. The synthetic function is conducted by the bone marrow, where both bone and blood cells are synthesized in a process called hematopoiesis [10]. Metabolic functions are related to act as a reservoir of calcium, phosphorus, growth factors and fat [11]. Besides, bone tissue helps to regulate pH level of blood releasing alkaline salts [12].

Referring to mechanical function, bones are the structural elements of the human body. Skeletal system supports loads due to the different activities of an individual as holding things, walking, pushing, and so forth. These loads induce tensile, compressive, or shear stresses on the bone tissue. More complex stresses such as those caused by bending or twisting of bone can be decomposed into the three basic aforementioned stresses. To study these stresses, bone mechanical properties such as elasticity modulus, compressive, and tensile strength are important. These properties are highly dependent on the position of the bone and the condition of the individual. Besides, mechanical properties of bone vary depending on the load orientation with respect to the orientation of the tissue (anisotropy) and the speed to which the load is applied (viscoelasticity) [3, 13]. Reference [14] provides a good source of data and models of mechanical properties for different types of human bones. Some important mechanical properties are described in Table 1.

Another important physical property of osseous tissue is permeability that describes the porosity and interconnectivity of tissue. Permeability is estimated between 0.003–11 × 10^−6^ m^4^/N·s for trabecular bone in humans and 0.9–7.8 × 10^−11^ m^4^/N·s in cortical bone for canine and bovine animals [15]. A detailed explanation of permeability in bone can be found in [16, 17].

Bone tissue may suffer various diseases that can be caused by excessive load or hormonal deficiencies, among other reasons [18, 19]. Bone tissue as an engineering material can fail because mechanical loads originate stresses over the limits a healthy bone can bear or because the mechanical properties of bone are decreased by various pathologies making the bone weak and prone to be damaged. Some of the diseases of bone tissue are as follows.

- Fracture: it is partial or total loss of bone continuity. It is caused by traumas by mechanical loads that exceed the allowable stresses of the bone. There may be associated factors to the extent that allowable stresses are conditioned by other diseases that affect bone density. They can be classified considering the type of trauma, fracture shape, and the location and direction of the load [20].
- Osteogenesis imperfecta: it is bone embrittlement due to deficiencies in the collagen matrix [21].
- Osteoporosis: it is loss of bone minerals by hormonal deficiencies [22].
- Osteomalacia or rickets: it is loss of bone mineral caused by nutritional deficiencies [23].
- Osteomyelitis: it is bone infection caused by bacteria [24, 25].
- Cancer: primary or metastatic type causes progressive damage of bone tissue and its functions [26].

As mentioned above, those diseases affect multiple demographic groups according their socioeconomic conditions. For example, in developed countries the life expectancy of the population has increased considerably causing a rise in osteoporosis cases [27].

## 2. Bone Tissue Engineering

Tissue engineering combines the use of cells, engineering materials, and physicochemical factors to improve or replace the biological functions of damaged tissues or organs. It uses the principles and methods of engineering, biology, and biochemistry for understanding the structure and function of normal and pathological mammalian tissues and for developing biological substitutes in order to restore, maintain, or improve its function [28]. A wide area of interest for tissue engineering is the development of scaffolds that contribute to bone regeneration processes [29]. This development could follow some or all of the stages listed below [30]:

1. scaffold fabrication;
2. growth factor placement in the scaffold or damaged area;
3. seeding of an osteoblast population into the scaffold in a static culture (petri dish);
4. growth of premature tissue in a dynamic environment (spinner flask);
5. growth of mature tissue in a physiologic environment (bioreactor);
6. surgical transplantation of the scaffold;
7. tissue-engineered transplant assimilation/remodeling.

The number and the way that previous stages are combined give complexity to the bone regeneration processes in tissue engineering. For scaffold fabrication, factors like size, mass, porosity, surface/volume ratio, form, surface shape, and chemistry of the element to be manufactured and composition, structure, molecular weight, and molecular orientation of the biomaterial must be considered. For stages that occur in* in vitro* environments, variables like culture medium, pH, fluid flow, mechanical stimuli, temperature, origin of cells, number of cells, mobility of the cells, and cell activity affect the growth of new tissue. Finally, defect site, species, gender, age, inflammatory process, immunological process, mechanical stimuli, biochemical stimuli, enzymes, and vascularization determine the bone regeneration processes in* in vitro* environments [15].

### 2.1. Socioeconomic Considerations

In 2007 it was calculated that the whole area of tissue engineering consists of 50 companies employing 3,000 equivalent full-time positions [31]. In 2010 the number of companies related to regenerative medicines was increased significantly to 391, but only a small portion of these has a commercial product [32]. About 500,000 bone grafts are performed each year in the United States [33]. This quantity is close to the estimation that between 5% and 10% of the 6 million fractures that occurred in North America present delays or consolidation problems in the healing process [34].

Scaffolds, implants, biomaterials, cell based therapies, and growth factors are usually considered as bone grafts substitutes in bone tissue engineering. Diverse analyses show different market sizes and their growth rates depending of what it is denominated as bone graft substitute: the global bone graft substitute market was valued at $1.9 billion in 2010 and it is forecast to reach $3.3 billion in 2017 [35]. Another source states that the market for orthopedic biomaterials in the United States was almost $3.4 billion in 2012 [36]. Another study affirms that 1 g of bone graft substitute costs approximately 100 USD and the volume of materials is estimated close to 10 tons per year in 2010 [37]. The European market for bone graft substitute products for spinal fusion was valued at USD 177 million in 2010 and its growth rate is projected close to 17% per year, reaching an estimated value of $461 million in 2016 [38]. The global bone graft substitute market consists of eight different segments [36]: orthopedic bone graft substitute, growth factors, stem cells, cell therapies, orthopedic hyaluronic acid viscosupplementation, orthopedic tendon graft, orthopedic cartilage repair, and spinal machined bone allograft. Growth factors represent the largest segment, close to 40% of the market. The segments related to synthetic materials represent only about 15% of the market, but their growth rate is the largest (close to 15% per year) [37].

The cost of replacing organs was estimated in 8% of the worldwide cost of health in 2009 [41]. The high cost of tissue engineering is associated not only with research and development but also with the regulations governing human healthcare products [42]. Besides, some reasons for the size and growth rates of the bone tissue engineering markets are an aging but more active population, the increase of overweight issues in population, the increased interest of individuals in their own healthcare, the improvement of public health systems around the world, and the development of orthopedic procedures for people of all ages [43].

### 2.2. Growth Factors

Growth factors are substances, like cytokines or hormones, which act as biochemical signals capable of triggering cellular processes like growth, proliferation, or differentiation, among others. The most considered growth factors in bone tissue engineering are listed below.

- Bone morphogenetic proteins (BMPs): BMPs are a family of cytokines that stimulates the proliferation of chondrocytes and osteoblasts and increases extracellular matrix production. BMPs induce the differentiation of mesenchymal stem cells into osteoblasts. BMPs allow not only skeletal tissue formation during embryogenesis, growth, and adulthood, but also bone healing process. In newborns' skeletons, BMPs can be found in the collagen fibers of the bone matrix and also in cells located in the periosteum and the bone marrow. After a fracture, BMPs growth factors diffuse from bone matrix and activate osteoprogenitor cells which, in turn, produce more BMPs [44]. The BMP 2, BMP 4, and BMP 7 are the only growth factors that can singly provoke bone formation in* in vitro* cultures and at* in vivo* heterotopic sites. BMPs 1–3 increase the production of collagen type I and osteocalcin in* in vitro* osteoblasts like cells and improve the formation of mineralized bone nodules from bone marrow mesenchymal stem cells [45]. BMPs are the most representative bone graft substitute of growth factors segment due to their therapeutic possibilities [31, 41, 46]. Studies of the combined application of BMPs and porous scaffolds indicate that these growth factors promote growth of new bone tissue inside these structures [47–51].
- Fibroblast growth factors (FGFs): FGFs stimulate the proliferation of mesenchymal cells, osteoblasts, and chondrocytes. FGFs enhance growth of different tissues due to their angiogenic properties. FGF-2 or bFGF is the most studied cytokine of this family for bone regeneration applications [51, 52].
- Insulin-like growth factors (IGFs): IGFs promote the proliferation of osteoblasts and chondrocytes and induce matrix secretion from both cell types [51]. IGFs stimulate collagen synthesis and mineralization of bone tissue [53].
- Platelet-derived growth factors (PDGFs): PDGFs increase the proliferation of chondrocytes and osteoblasts. However, depending on their concentrations levels, they have also been implicated in bone resorption [51]. PDGFs act as chemotactic and mitogenic factor for osteoblasts and other cells [54].
- Transforming growth factors-*β* (TGFs-*β*): TGFs-*β* cause the differentiation of mesenchymal cells into chondrocytes and may also induce chondrocyte and osteoblast proliferation [55]. Like PDGFs, they have been seen to increase bone resorption at certain concentrations playing a role in coupling bone formation and resorption activities [51].

### 2.3. Scaffolds

Scaffolds are fundamental devices for the regeneration of lost or damaged tissues and they have become an important tool in tissue engineering [56]. Their functions, from the mechanical point of view, consist of bearing external loads and giving shape to the tissue that is regenerated on it [57–59]. From the biological point of view, those structures support the development of extracellular matrix and cell colonization. In addition, scaffolds should allow transit of nutrient substances from the surrounding tissue or the culture media and waste disposal coming from the tissue being formed. Therefore scaffold stiffness, mechanical resistance, and permeability are important properties. An additional scaffolds' desirable feature may be a controlled degradation after they are implanted in order to get void space where new tissue can grow.

The mechanical properties and degradation of the scaffold depend on the material properties and the porosity geometry of its structure; meanwhile permeability depends on its structure. The mechanical properties of the scaffold must be similar to the properties of the replaced bone tissue in order to prevent stress shielding. Finally, the degradation rate must be as close as possible to the tissue growth rate to maintain stable properties in the tissue-scaffold compound during the regeneration process.

#### 2.3.1. Design Considerations

A bioactive scaffold reacts in a controlled manner with its environment in order to stimulate specific biological responses where it is placed. The development of scaffolds to promote cellular growth inside them has been one of the fundamental goals of bone tissue engineering [30, 60, 61]. The biomechanical processes of bone regeneration are complex, so the requirements for scaffold design are diverse [12, 62–68]. Some of the most important design considerations are listed below.

- Biofunctionality: it is ability of the scaffold to meet the functional requirements for which it was designed, restoring the functions of the replaced tissue.
- Biocompatibility: it is ability to support normal cellular activity including molecular signaling systems without eliciting or evoking local or systemic adverse effects to the host. Among the undesirable effects that must be eliminated, minimized, or controlled upon scaffold implantation in the body are cytotoxicity, genotoxicity, immunogenicity, mutagenicity, thrombogenicity, and swelling. For example, inflammation should be avoided because it can decrease the regeneration rate and promote tissue rejection.
- Bioresorbability or biodegradability: it is ability to degrade with time in* in vitro* or* in vivo* environments, preferably at a controlled resorption rate in order to create space for new tissue to grow. In other words, it is expected that, as long as cells proliferate, void space in the scaffold increases and degradation rate of the material should match growth rate due to healing or regeneration process. It is related with biocompatibility because degradation products should be nontoxic and must be able to get metabolized and eliminated from the body. For example, the degradation behavior of the scaffolds should vary based on applications such as 9 months or more for scaffolds in spinal fusion or 3–6 months for scaffolds in craniomaxillofacial applications [69].
- Mechanical properties: mechanical properties such as elastic modulus, tensile strength, fracture toughness, fatigue, and elongation percentage should be as close as possible to the replaced tissue (mechanical compatibility) in order to prevent bone loss, osteopenia, or “stress shielding” effect associated with the use of bone grafts. They are related to bioresorbability because the variation in mechanical properties due to degradation process should be compatible with bone regeneration process. A scaffold must have enough mechanical strength to retain its structure in order to comply with its mechanical function after its implantation in the case of hard, load-bearing tissues as bone. The large variation in mechanical properties as seen in Table 1 makes it difficult to design an “ideal bone scaffold.”
- Pore size and porosity: a three-dimensional design affects the spatial distribution and location of cells, nutrients, and oxygen, thus affecting the viability of the new formed tissue. Porous scaffolds facilitate the migration and proliferation of cells, providing an appropriate microenvironment for cell proliferation and differentiation and allowing the mass transfer of nutrients, oxygen, and waste metabolic products within the structure. Scaffolds should have a large internal surface area due to overall porosity and pore size. The surface to volume ratio of porous scaffolds depends on the size of the pores. A large surface area allows cell adhesion and proliferation, whereas a large pore volume is required to contain and later deliver a cell population sufficient for healing or regeneration process. Mass transfer and cell migration will be inhibited if pores are not connected even if the overall porosity is high. Unfortunately, an increase in porosity causes a decrease of mechanical properties such as compressive strength and increases the complexity for scaffold manufacturing. On the other hand, osseous tissues typically have arranged on curved surfaces; therefore, to mimic this biomorphic pattern, pores are intended to have curved cross sections [12].

Comprehensive lists of terms related to tissue engineering and biomaterial are available in [70, 71]. With regard to bone scaffolds, there are some specific features like the following.

- Osteoconductivity: it is ability to allow the bone cells to adhere, proliferate, and form extracellular matrix on its surface and pores [69]. This property is related to the biodegradability because the scaffold material must be reabsorbed to make space for the mature tissue that it initially helped to support. Besides, scaffolds act as a mold of the desired anatomical form.
- Osteoinductivity: it is ability to induce new bone formation through biomolecular or mechanical stimuli, recruiting progenitor cells and allowing differentiation in a controlled phenotype or particular lineages [72].
- Osteogenicity: it is ability to act as osteoblasts or mesenchymal cells (capable of deriving in an osteoblastic lineage) reservoir because these cells can form and mineralize the extracellular matrix of new osseous tissue.
- Osteointegrity: it is ability to form strong bonds with surrounding osseous tissue allowing material continuity and proper transfer load.

Finally, additional functions for bone scaffolds could be as follows [73–75]:

- acting as carrier of drugs (i.e., antibiotics and/or anti-inflammatories), growth factors, or cultured cells;
- radiolucency: ability to differentiate radiographically with respect to the tissue where it was implanted;
- formability: ability to be shaped by a manufacturing process in order to obtain the necessary internal and external geometry;
- sterilizability: ability to ride out and facilitate a process of microbial destruction after being manufactured and before being used;
- stability on storage (shelf life): ability to preserve the physical, chemical, and dimensional properties within the estimated storage period between manufacture and its use.

The conflicting nature of the above desired characteristics was described by Karageorgiou and Kaplan [76] who reported that higher porosities induce greater bone ingrowth but lower mechanical stiffness and strength. Therefore, scaffold porosity must lie within a critical range small enough to maintain the mechanical integrity of the scaffold and large enough to provide optimal bioactivity [63].

#### 2.3.2. Design Scales

The design and fabrication of scaffolds for bone regeneration applications attempt to obtain and control architecture at different levels due to external form and internal structure to meet the clinical requirements specified in the previous section. The architecture has different properties and characteristics depending on the dimensions of an element of the scaffold. Three basic scales refer to different features and processes.

The macro-mesoscale describes the geometry measured in millimeters. Among its features are the following [77–82]:

- scaffold external shape (appropriated to the site where it will be implanted);
- mechanical properties;
- density;
- porosity: as a percentage of volume of the scaffold is empty.

The microscale describes features in the order of micrometers as

- pore size;
- interconnectivity of pores and tortuosity;
- degradability.

The features in nanometers include factors such as

- surface topology of the pores;
- surface physical chemistry.

#### 2.3.3. Porosity Design

Pore size and porosity are important geometric properties in scaffolds for bone regeneration because they affect the phenotype and the amount of tissues that grow on the construct. As mentioned before, interconnected pores are necessary for bone tissue regeneration because they allow migration and proliferation of osteoblasts and mesenchymal cells besides vascularization. It is observed that even a biomaterial like hydroxyapatite must have a porous structure in order to promote bone growth* in vivo* [83] or a high porosity to allow cell seeding* in vitro* [84]. Scaffolds implanted* in vivo* with pore sizes close to 100 *μ*m allow chondrogenesis but scaffolds with pores close to 350 *μ*m promote osteogenesis [85]. Although intensive research has been developed in both experimental and computational modeling, there are no final conclusions about the optimal porosity and pore size of a scaffold for bone regeneration. For example, the porosity range is between 50 and 90% for scaffolds that are not subjected to mechanical loads [86]; meanwhile the recommended size of the pores varies between 150 and 600 *μ*m [87], from 400 to 1200 *μ*m [88] and 350 *μ*m or above [76]. The variety of conclusions may be due to the complexity of the process of bone regeneration, which is multivariable and multiobjective [89].

Considering this and the emergence of solid-free form manufacturing methods to fabricate scaffolds [90] that allow controlling geometry characteristics better than other conventional methods like salt leaching there is an increasing interest in porosity design. Giannitelli et al. showed an extensive review of the design techniques used to create porous structures in the scaffolds noting that these geometries can be obtained in three ways [67]: periodic structures, nonperiodic structures, and optimization techniques. Periodic porous structures can be based on CAD systems for solid and surface modeling, such as constructive solid geometry (CSG) using primitives like cubes, cylinders, and spheres to represent the pores [91–97] and boundary representation (B-Rep) supported on facets and vertices [98]. In the last years, there is research about the use of implicit surfaces like triply periodic minimal surfaces [99–104] and space-filling curves like Hilbert curves [105]. Meanwhile, nonperiodic structures have been developed based on image of bone surfaces [77], trabecular bone portions [106, 107] or scaffolds [108], and stochastic methods and Voronoi diagrams [109]. The disadvantage of periodic and nonperiodic structures is the necessity of trial and error methods to determine if they are suitable for a particular purpose [110, 111]. In contrast, optimization methods [112] using finite element methods obtaining porous structures considering different objectives as mechanical properties and permeability [59, 60, 113–115].

## 3. Biomaterials for Bone Tissue Engineering

A number of definitions have been developed for the term “biomaterials.” One definition is “material exploited in contact with living tissues, organisms, or microorganisms” [71]. Another definition is as follows: “a biomaterial is a substance that has been engineered to take a form which, alone or as part of a complex system, is used to direct, by control of interactions with components of living systems, the course of any therapeutic or diagnostic procedure, in human or veterinary medicine” [116]. In general, biomaterials are intended to interface with biological systems to evaluate, treat, augment, or replace any tissue, organ, or function of the body and are now used in a number of different applications throughout the body. The major difference of biomaterials from other classes of materials is their ability to remain in a biological environment without damaging the surroundings and without being damaged in that process.

Naturally derived materials, ceramics, polymers and composites can be used as biomaterials. Natural biomaterials can be the bone from the same individual (autografts), from individuals of the same species (allografts) or from different species (xenografts). On the other hand, ceramic materials are based on calcium phosphates and bioglasses. They have good osteoinductive properties but low mechanical properties and difficulties in forming process. Polymers such as those derived from polyglycolic acid (PGA) and polylactic acid (PLA) have easy formability, good mechanical properties and biodegradability which may vary according to their molecular weight but low osteoinductive capacity. For their part, ceramic-polymer composite materials allow obtaining a biodegradable material, with good mechanical strength, osteoinductive, osteoconductive, and conformability properties combining the properties of each material family. Here we mention some of them.

### 3.1. Grafts

A biomaterial commonly used for bone regeneration is osseous tissue taken from the same individual (Autografts). Autografts are considered the “gold standard” because they are osteoinductive, osteoconductive, and osteogenic. This material is normally taken from a site that is not under mechanical load such as the iliac crest. Autografts contain cells and growth factors that support the process of bone regeneration and do not exhibit risk of rejection and disease transmission [117]. Some drawbacks of autografts are the necessity of additional surgeries, possible infections, morbidity of the bone, pain, and its limited availability. Depending on the source of the osseous tissue there may also be allografts (tissue from individuals of the same species) or xenografts (tissue from individual of different species). Allografts presented benefits as ready availability and easy handling but require treatments such as freeze drying, irradiation, and washing with acid, among others, to prevent rejection by the receptor and remove any possible infections from the tissue to be implanted; these processes can affect their mechanical and biological properties. Xenografts that usually come from cows and coral [118] could be osteoinductive and osteoconductive and with low cost with high availability but have the disadvantages of immune response and risk of transmission of animal diseases [119].

### 3.2. Ceramics

Ceramic materials are a group of inorganic oxides and salts used in bone tissue engineering because of their similarity to the mineral component of bone in the case of calcium phosphate or because of their capacity of strength bonding to osseous tissues in the case of bioglasses [117]. Some ceramic materials used in bone regeneration are listed below.

*Calcium Phosphates*. Calcium phosphates are a family of minerals composed of calcium ions (Ca^2+^), orthophosphates (PO_4_^3−^), metaphosphates or pyrophosphates (P_2_O_7_^4−^), and sometimes hydrogen or hydroxide ions. The most common calcium phosphates for tissue engineering are hydroxyapatite (HA), calcium sulphate hemihydrate (CSH), gypsum, calcium sulphate dehydrate (CSD), calcium carbonate, dicalcium phosphate (DCP), octacalcium phosphate (OCP), *β*-tricalcium phosphate (*β*-TCP), biphasic calcium phosphate (BCP), and *β*-calcium pyrophosphate (*β*-CPP) [37]. Commercially available calcium phosphates proceed from natural or synthetic sources and are processed in many physical forms like particles, blocks, cements, and coatings on metal implants or composites with polymers.

The most common calcium phosphate for bone tissue regeneration is hydroxyapatite (HA) which is a crystalline calcium phosphate (Ca_10_(PO^4^)_6_(OH)_2_) present in bones. Depending on its source, it can be natural or synthetized, for example, it can be produced from calcium carbonate and monoammonium phosphate at ambient pressure [120] or from natural sources like cattle or coral [121]. Some HA presentations exhibit a very similar bone structure with osteoconductive characteristics allowing connective tissue surrounding and start the regeneration process.

Calcium phosphates are bioactive materials because of their ability to form bone apatite like material or carbonate hydroxyapatite on their surfaces. They have the ability to promote cellular function and expression besides the capacity of forming a strong bind between bone and biomaterial interface. In addition, calcium phosphates biomaterials processed in porous forms are capable of binding and collecting growth factors and become osteoinductive biomaterials [122, 123]. In addition, calcium phosphates are materials that allow adhesion of osteoblasts and promote mesenchymal cells migration. Related to degradation, tricalcium phosphates are capable of tunable bioresorption rate [124]. Different calcium phosphates can be used simultaneously to improve the scaffold performance [125].

Calcium phosphates applications in bone regeneration include their use as a scaffold in periodontal treatment, healing of bone defects, fracture treatment, total joint replacement, orthopedics, craniomaxillofacial reconstruction, and spinal surgery. Moreover, calcium phosphates are widely applied as a coating material to provide strength to polymeric scaffolds or to enhance the bioactivity on metal surfaces [121].

*Bioglasses*. Bioglasses are a family of bioactive glasses, compound of SiO_2_, Na_2_O, CaO, and P_2_O_5_ in variable proportions. There are several types of bioactive glasses: conventional silicates, such as bioglass 45S5, phosphate-based glasses, and borate-based glasses. A hydroxycarbonate apatite (HCA) layer is formed on the surface of the glass, following initial glass dissolution. HCA is similar to bone mineral and interacts with collagen to bind the bioglass with the host tissue. Osteoinductivity in bioglasses is related to the action of dissolution products of these biomaterials on osteoprogenitor cells stimulating new bone growth. Besides, the HCA layer provides a surface capable of enhancing osteogenic cell attachment and proliferation. As calcium phosphates, the HCA layer adsorbs protein and growth factors to promote new bone formation. An advantage of bioglasses above calcium phosphates is their faster degradation rate [126].

Bioglasses are used in bone regeneration like periodontal pocket elimination, alveolar ridge augmentation, maxillofacial reconstruction, spinal surgery, and otorhinolaryngological reconstruction [127, 128]. They can be processed and manufactured to generate a range of three-dimensional scaffolds with different porosities and surface characteristics [117].

### 3.3. Polymers

In tissue engineering, biopolymers are synthetic organic materials which are biocompatible with humans. They may be of natural or synthetic origin. Among the natural polymers used for tissue regeneration are those materials inspired by the extracellular matrix like collagen [129]. Among the synthetic polymers used for bone tissue regeneration are polylactic acid (PLA), polyglycolic acid (PGA), and copolymers of PLA-PGA (PLGA). Properties of some polymers and copolymers biomaterials are listed in Table 2. A detailed list of commercial polymeric scaffolds' products can be found in [130, 131].

Collagen: collagen is the main component of connective tissue in mammals. Collagen type I is present in the form of elongated fibrils in bone and is the most abundant in nature and most considered for biomedical applications. It possesses good biocompatibility and low antigenicity. Collagen has the ability of crosslinking; therefore, mechanical and degradation properties can be tailored [129]. Collagen type I has Young's modulus of 5 ± 2 GPa for dry fibrils and from 0.2 to 0.5 GPa for fibrils immersed in phosphate-buffered saline (PBS) [132].

Poly(*α*-ester)s: poly(*α*-ester)s are thermoplastic polymers with hydrolytically labile aliphatic ester bonds in their chains. Poly(*α*-ester)s can be developed from a variety of monomers using ring opening and condensation polymerization routes changing the monomeric units. Bioprocess methods can be used to develop some poly(*α*-ester)s [133]. The poly(*α*-ester)s are biodegradable, nontoxic, and biocompatible. Among poly(*α*-ester)s, the most extensively investigated polymers are the poly(*α*-hydroxy acid)s, which include poly(glycolic acid) and poly(lactic acid). The most extensively studied monomers for aliphatic polyester synthesis for biomedical applications are lactide, glycolide, and caprolactone [134]. Poly(*α*-ester)s mainly are degraded by hydrolysis bulk erosion. The polymeric matrices degrade over their all cross section and have erosion kinetics that usually are nonlinear with discontinuities [135].

Polyglycolide (PGA) is a highly crystalline polymer (45–55% crystallinity); therefore, it exhibits a high tensile modulus with very low degradation rate due to organic solvents. The first biodegradable synthetic suture that was approved by the FDA in 1969 was based on polyglycolide [136]. Nonwoven polyglycolide scaffolds have been widely used as matrices for tissue regeneration due to their excellent degradability, good initial mechanical properties, and cell viability. High mechanical properties of PGA are due to its high crystallinity. Self-reinforced forms of PGA show higher stiffness than other degradable polymeric systems used clinically and exhibit an elasticity modulus of approximately 12.5 GPa. Polyglycolide degrades by nonspecific scissions of the ester chain. PGA loses its strength in 1-2 months when hydrolyzed and losses mass within 6–12 months. In the body, PGA degradation product is glycine which can be excreted in the urine or converted into carbon dioxide and water via the citric acid cycle [137]. Due to its good initial mechanical properties, polyglycolide has been investigated as bone internal fixation devices (Biofixs). However, the high rates of degradation and acidic degradation products limit the clinical applications of PGA. Therefore, copolymers containing PGA units are being developed to overcome those disadvantages.

Polylactide (PLA) is a chiral molecule and exists in two optically active forms: L-lactide and D-lactide. Their polymerization forms a semicrystalline polymer and PLA behaves as crystalline or amorphous depending of these stereoisomers. The polymerization of racemic (d,l)-lactide and mesolactide results in the formation of amorphous polymers [138, 139]. The molar mass of the polymer as well as the degree of crystallinity has a significant influence on the mechanical properties [140].

Poly-L-lactide (PLLA) is a low rate degradation polymer compared to PGA and has good tensile strength and high Young's modulus (4.8 GPa approx.); therefore, it is useful for load-bearing applications, such as orthopedic fixation devices [134]. It has been reported that high molecular weight PLLA can take between 2 and 5.6 years for total resorption* in vivo* [144]. On the other hand, semicrystalline PLA is selected to the amorphous polymer when better mechanical properties are necessary. Semicrystalline PLA has an approximate tensile modulus of 3,5 GPa, tensile strength of 50 MPa, flexural modulus of 5 GPa, flexural strength of 100 MPa, and an elongation at break of about 4% [145].

Poly(lactide-co-glycolide) (PLG): both L- and DL-lactides have been used for copolymerization with glycolide monomers in order to obtain different degradation rates. PLG degradation rates depend on a variety of parameters including the LA/GA ratio, molecular weight, and the shape and structure of the matrix. For example, 50/50% poly(DL-lactide-co-glycolide) degrades in approximately 1-2 months, 75/25% PLG in 4-5 months, and 85/15% copolymers in 5-6 months. [146]. The popularity of these copolymers can be attributed to the FDA approval for use in humans and their good processability [134].

Polycaprolactone (PCL): PCL is semicrystalline polyester obtained by the ring opening polymerization of monomeric units of “*ε*-caprolactone.” PCL presents hydrolytic degradation due to the presence of hydrolytically labile aliphatic ester bonds; however, the rate of degradation of homopolymer is rather slow (2-3 years) with respect to polymers like PLA. PCL has low tensile strength (approximately 23 MPa) and high elongation at breakage (>700%) [134]. It can be used in conjunction to other materials for load-bearing applications [147].

### 3.4. Biocomposites

The literature review shows in recent years a trend in the development of scaffolds made of ceramic/polymer composites [142]. This is because ceramics like calcium phosphates have excellent osteoinductive properties but low degradability, low mechanical strength, and difficulty in forming processes for controlling the physical and geometrical characteristics required from the scaffold. Furthermore, polymers such as PLA exhibit poor osteoinductivity but better mechanical properties and degradability rates besides that they can be formed by various manufacturing processes that allow better control of their geometric characteristics. Composites of collagen type I and calcium phosphates are widely used in bone tissue engineering due to the similarity to natural bone and capacity of enhancing osteoblast differentiation and accelerating osteogenesis [143, 148, 149]. The development of ceramic-polymer composites allows biodegradable materials with good mechanical and biological properties as seen in Tables 3 and 4.

### 3.5. Biomaterial Degradation

In the case of scaffolds made of biodegradable polymers, many resorption mechanisms are identified depending of the material type [37]. In those models water molecules diffuse into the polymer and break the link into polymer molecules. This phenomena cause a molecular weight decrease besides a decrease of elasticity modulus. After a certain threshold of molecular weight the polymer is considered completely degraded [57, 135]. A more elaborate model is proposed by Chen et al. [78] including autocatalysis. Han proposed a model that includes the effect of crystallization [160]. On the other hand, ceramics such as calcium phosphates and hydroxyapatite degrade by dissolution and osteoclasts effect as modeled in [161] (Table 5).

### 3.6. Scaffold Fabrication Techniques

Various manufacturing methods have been used to achieve certain properties at different scales. These methods are classified into conventional and additive manufacturing methods. Conventional methods are solvent casting/particulate leaching, phase inversion/particulate leaching, fiber meshing/bonding, melt molding, gas foaming, membrane lamination, hydrocarbon templating, freeze drying, emulsion freeze drying, solution casting, and ceramic sintering. These methods use physicochemical phenomena to ensure internal structures with a variable pore size between 100 and 500 microns with porosities up to 90% [15]. They have the disadvantage that internal structure consists of randomly arranged trabeculae and physical properties as permeability vary and are difficult to control. In recent years, methods of additive manufacturing, also called rapid prototyping (RP) or solid free-form modeling (SFF), have been used for scaffold fabrication. Some of these methods are fused deposition modeling (FDM), three-dimensional printing or plotting (3DP), selective laser sintering (SLS) and stereolithography (SLA). These methods achieve large scaffolds with oriented structures but fail to obtain high porosity with small pores. Detailed lists of specific materials, processing methods, and properties obtained are given in [15, 130]. On the other hand, an alternative to solid bone scaffolds is injectable bone cements [162, 163]. These are mainly used in the fixation of prostheses and filling bone cavities and kyphoplasty treatments [164].

## 4. Mechanobiology of Bone Tissue

Mechanobiology studies show how mechanical stimuli influence the shape and structure of tissues of living beings, in particular, muscle, tendon, cartilage, and bone tissues [165]. Mechanical and biochemical stimuli influence proliferation, differentiation, and cell functions [166]. Therefore, mechanobiology would be useful to suggest clinical and tissue engineering strategies to control osseous tissue behavior.

Bone tissue is formed by a process called osteogenesis [167]. In this process, cells capable of producing tissue interact with chemotactic factors to form bone. Firstly, osteoblasts secrete substances to form osteoid tissue or immature bone, a nonmineral matrix compound of collagen [168] and glycosaminoglycans. Subsequently, the matrix mineralization occurs by deposition of hydroxyapatite [169, 170]. During this process, some osteoblasts become trapped in the newly formed bone and become osteocytes surrounded by osteons. Osteocytes maintain the extracellular matrix and it is hypothesized that they act as a network sensing mechanical stimulus that activates the bone remodeling units (BMUs) formed by osteoblasts and osteoclasts.

Once the bone is formed, it can be remodeled or regenerated by mechanical and biochemical stimulus. Remodeling process took place in old bone when tissue is replaced by new one in order to support changing loads or to replace bone with microdamage. A turnover rate of 100% per year in the first year of life, 10% per year in late childhood [12], and near 5% per year in adult life [171–173] is estimated. Regeneration allows the creation of new tissue when an injury or lack of continuity occurs, for example, in case of fracture [174–176]. Both processes are carried out by BMUs [177–180], in which osteoclasts resorb deteriorated bone matrix and osteoblasts deposit new bone. Sometimes, those processes present disorders like in Paget's disease [181]. The processes of remodeling and regeneration are still under study because of the large number of physical and biological factors creating complexity in their interactions [13]. For example, it is hypothesized that osteocytes by piezoelectric phenomena respond to mechanical deformations or stresses and send signals to osteoblasts and osteoclasts so they engage and conform BMUs to perform the resorption or deposition of new bone [2].

Remodeling and regeneration require actions at different scales. The mechanosensitive/mechanoresponsive process starts in nanoscale or molecular level activating genes and signals in cells [182, 183], and it continues with a mechanotransduction process at cell level in nano-microscale activating electrical, chemical, or biochemical activity, for example, ion channels or integrins, the differentiation of mesenchymal cells into bone cells (osteocytes, osteoblasts, and osteoclasts), and the interactions of those cells in the bone deposition and resorption processes [184]. Finally, in a macroscale, stimuli determine the mechanical properties of bone tissue, bone shape, and magnitude of the loads they can support. One example of adaptation of shape and structure of bone due to mechanical loads is described in Wolff's law [185–187]. It states that bone adapts its internal and external form depending on the forces applied on it [188].

From the clinical point of view, mechanobiology is studied using* in vivo* and* in vitro* models. These methods can be expensive, time-consuming, and difficult to control and, in some cases, with ethical drawbacks. An alternative to these models are computational methods or* in silico* experiments. Computational mechanobiology studies the effect of mechanical stimuli in the differentiation, growth, adaptation, and maintenance of tissues, establishing qualitative and quantitative rules between the different variables involved in these processes. In computational mechanobiology numerical methods, generally finite element method, FEM, are used to solve systems of equations describing the relationships between the variables and parameters of the phenomena studied. Whereas some variables and parameters of these processes may not be measurable, trial and error methods are applied [189, 190].

### 4.1. Mechanical Stimuli Variables

A first task in computational mechanobiology is to determine which mechanical stimulus will serve as input variable. The mechanical stimuli that monitor the cells and the means they used to measure that signal are still debated [191–196]. Signals can be essentially volumetric deformation component (change in size) and a deviatoric deformation component (change in shape). Several researchers have proposed various types of mechanical signals: Frost proposed a minimum stress value in the osseous tissue to trigger a bone apposition process [197], and later, he changed the stress signal by a deformation signal [198, 199], Carter et al. propose the principal strain and hydrostatic stress as mechanical signal [200], Claes and Heigele use the principal strain and the hydrostatic pore pressure to study the fracture healing process [201], Lacroix and Prendergast use the deviatoric strain and fluid velocity to study tissue differentiation in fracture healing [202], and Huiskes et al. studied strain energy density or SED to predict bone remodeling [203]. The output variables help to describe the differentiation process (how many and which cell lines are produced), proliferation (which is the rate of growth), and adaptation and maintenance of tissues (position and mechanical properties of formed tissues).

### 4.2. Regeneration and Remodeling of Bone Tissues

**Figure 1:**
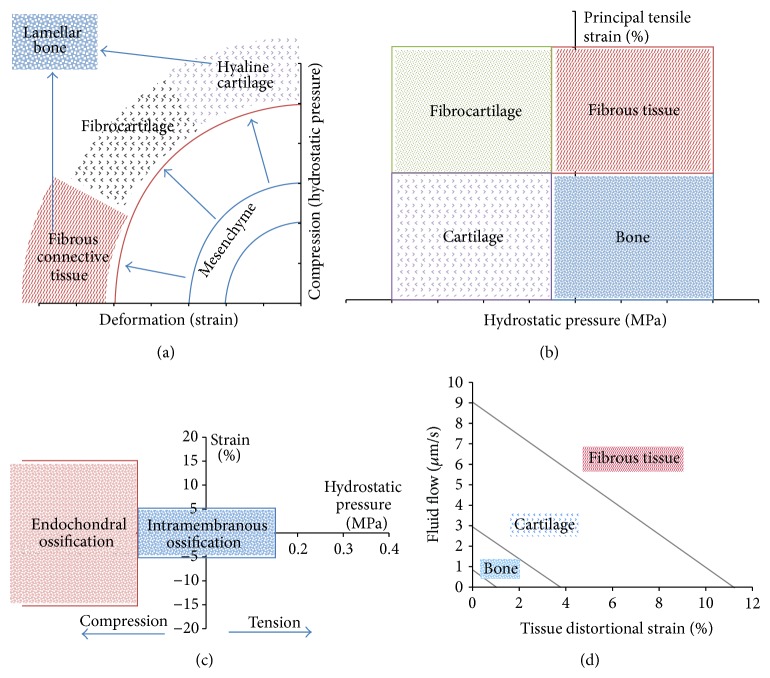
Graphic representation of mechanoregulatory models proposed by (a) Pauwels [204], (b) Carter et al. [200], (c) Claes and Heigele [201], and (d) Prendergast [205]. Adapted from Geris et al. [34] with permission from the Royal Society.

The study of the bone regeneration process may consider tissue differentiation depending on the type and magnitude of the mechanical stimulus. There are four basic mechanoregulatory models of bone tissue differentiation. Pauwels postulated that high strains led to the formation of fibrous tissue, while higher pressures led to cartilage tissue [204]. Later, Carter et al. proposed a model where the type of tissue depends on the direction and magnitude of the stress. For example, the osseous tissue is possible where stresses and deformations have low magnitudes due to tension [200]. Claes and Heigele [201] developed a model that, unlike the previous two qualitative models, proposes ranges of values in which different types of tissues are obtained. For example, osseous tissue is generated by intramembranous ossification if the stress is ±0.15 MPa and the strain is less than ±5%. Finally, Lacroix and Prendegrast propose a model where tissues are not considered as a single material but as solid phase biphasic poroelastic materials. In this model, high fluid velocity values and deviatoric strains cause fibrous tissue [205]. Those models are represented in Figure 1.

Another line of research involves bone remodeling. This process includes the adaptation of the properties of the tissue that supports the mechanical loads. This line of work, developed by Fyhrie and Carter [206], has been extensively used in computational models. Here, bone tissue is considered as a continuous system with variable apparent density (*ρ*). This apparent density is expressed in terms of the stress (*σ*) to which the material is subjected. This is defined by the expression. It is considered that the bone tissue is a continuous system with variable apparent density *ρ*. This apparent density is expressed in terms of the stress *σ* to which the material is subjected. This is defined by the expression

(1) $$\begin{array}{c} \rho =A\sigma^{\alpha }, \end{array}$$

where *A* and *α* are constants. Considering *α* = 0.5, it follows that

(2) $$\begin{array}{c} \sigma^{2}=2EU, \end{array}$$

where *E* is the elastic modulus and *U* is the strain energy density. With regard to the elastic modulus *E*, experimentation leads to the relationship

(3) $$\begin{array}{c} E=c\rho^{3}. \end{array}$$

For example, one form of this equation that considers the viscoelastic behavior of the material is

(4) $$\begin{array}{c} E_{\text{axial}}=C{\dot{\varepsilon }}^{0.06}\rho^{3}, \end{array}$$

where *C* is a constant that considers values of elastic modulus and density of reference while $\dot{\varepsilon }$ is the rate of deformation.

Therefore, considering that bone remodeling is an optimization problem follows that the strain energy and bone density are related by

(5) $$\begin{array}{c} \rho =c^{\prime }U. \end{array}$$

### 4.3. Other Processes

Besides bone regeneration and bone remodeling due to mechanical stimulus another processes must be considered in bone tissue engineering. Sengers et al. [207], in an extended review, analyze the processes listed below.

- Proliferation: it is growth of cell population due to mitosis. Exponential or logistic law is usually considered here [153, 155].
- Nutrient transport and consumption: they are nutrient concentrations gradients due to cell population location and generation and disposal of waste substances. Regarding the interaction of nutrient availability and cell proliferation reaction diffusion equations is employed as seen in [153, 155].
- Senescence: it is decrease of cell population due to apoptosis [184, 208].
- Motility: it is cells movement and adhesion throughout their environment due to taxis. Although in bone remodeling and regeneration process it is usually considered that osteoblasts are not migrating cells, models as random walk or diffusion sometimes are applied. Random walk is a stochastic process that consists of a series of discrete steps of specific length. A random variable determines the step length and walk direction [63, 209]. Diffusion processes are used to predict osteoblast movement [210] or Darcy's law to model movement in porous media [154].
- Differentiation: stem cells turn into other more specialized cell types. Regarding bone regeneration, mesenchymal cells turn into fibroblast, chondrocytes, and osteoblasts not only due to mechanical signal as mentioned above but also due to chemical factors.
- Extracellular matrix changes: cells like osteoblasts produce matrix components (i.e., collagen and hydroxyapatite) and matrix degradation may occur by the action of osteoclasts.
- Cell to cell interactions: cells can communicate with each other in order to trigger processes. For example, osteocytes act as receptors of mechanical or chemical signals and dispose the formation of BMUs.

### 4.4. The Mechanostat Theory

Frost suggests that bone change must be considered in two phases: the internal phase, where the bone tissue changes its density and so its mechanical properties and the external one where there are changes due to the deposition or removal of osseous tissue on the bone surface [197]. In both cases the remodeling process is active depending on the value of the mechanical stimulus. It can be seen that in a range of mechanical stimuli, remodeling is inactive [203].

For external remodeling, the rate at which a bone is deposited or removed is given by

(6) $$\begin{array}{c} \frac{dX}{dt}=C_{x}\left(U-U_{n}\right), \end{array}$$

where *X* is the thickness of the formed layer, *U* is the strain energy density (SED), *U*_*n*_ is a reference value, and *C*_*x*_ is proportionality constant. Similarly, Young's modulus *E* change due to the mechanical stimulus is

(7) $$\begin{array}{c} \frac{dE}{dt}=C_{E}\left(U-U_{n}\right). \end{array}$$

**Figure 2:**
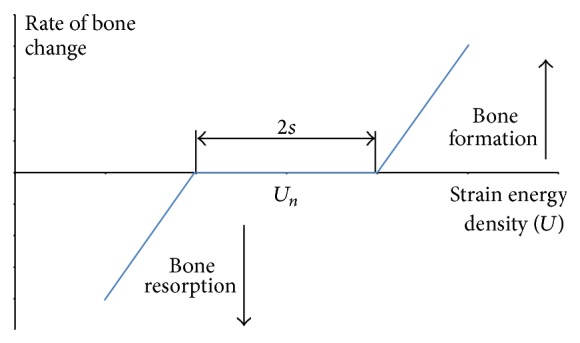
Rate of bone change as a function of the strain energy density (*U*). From Frost [197].

Therefore, the description of external remodeling process (Figure 2) is given by

(8) hhhhhhhhdXdt=CxU−1+sUnfor  U>1+sUn  Bone  formation0 for  1−sUn>U>1−sUn  Lazy  zonehhhhhhhhdXdt=CxU−1−sUnfor  U>1+sUn  Bone  resorption.

Meanwhile, the elasticity modulus change for internal remodeling is expressed as

(9) hhhhhhhhhdEdt=CEU−1+sUnfor  U>1+sUn  Stiffness  increase0 for  1−sUn>U>1−sUn  Lazy  zonehhhhhhhhhdEdt=CEU−1−sUnfor  U>1+sUn  Stiffness  decrease.

This type of response model to mechanical stimulation is widely used in bone remodeling and bone regeneration simulations.

### 4.5. Adaptive Remodeling with Variable Loading Conditions

A drawback of the models previously discussed is that they do not propose how to consider the effect of variable loads. Jacobs et al. [211] suggest a model that considers the effect as

(10) $$\begin{array}{c} \psi_{b}={\left(\overset{n}{\underset{i=1}{\sum }}n_{i}{\bar{\sigma }}_{b}\right)}^{1/m}, \end{array}$$

where *n* is the number of load cases considered, *n*_*i*_ is the number of times that the load is applied per day, and ${\bar{\sigma }}_{b}$ is the average cyclic stress. On the other hand, the work of Carter et al. [212] contributes to bone remodeling models weighting the effect of various loads. The expression for the mechanical stimulus *S* is

(11) $$\begin{array}{c} S=\frac{1}{n}\frac{1}{\rho }\overset{n}{\underset{i=1}{\sum }}U_{i}, \end{array}$$

where *U*_*i*_ is the mechanical stimulus for the *i*th load case, *n* is the total number of load cases, and *ρ* is the apparent density. Weinans et al. [213] used this stimulus to establish the change in bone bulk density by

(12) $$\begin{array}{c} \frac{d\rho }{dt}=B\left(S-k\right), \end{array}$$

where *S* is the stimulus with *B* and *k* as constants.

### 4.6. Bioinspired Models

In contrast to previous models, Mullender and Huiskes [214, 215] model the action of osteoblasts and osteoclasts separately. It is considered that the process of bone remodeling in a location *x* at time *t* is given by

(13) $$\begin{array}{c} \frac{dm_{\text{tot}}}{dt}=\frac{dm_{\mathrm{cl}}\left(x,t\right)}{dt}+\frac{dm_{\text{bl}}\left(x,t\right)}{dt}. \end{array}$$

The variables *m*_cl⁡_ and *m*_bl_ represent the adsorbed material by osteoclasts and deposited material by osteoblasts, respectively.

The second term represents the material apposition, which is given by

(14) $$\begin{array}{c} \frac{dm_{\text{bl}}\left(x,t\right)}{dt}=\tau \left(P\left(x,t\right)-k\right), \end{array}$$

where *P*(*x*, *t*) is the stimulus received by the osteoblast from the osteocyte, *k* is a reference value for the stimulus, and *τ* is a constant of proportionality. It should be noted here that the stimulus is not the value of the strain energy density at the point considered; it is the weighted summation of signals sent by osteocytes in the neighborhood around that point. Ruimerman et al. [216] applied this model to simulate bone adaptation under the influence of changes in the load orientation.

### 4.7. Mathematical Modeling of Bone Regeneration on Scaffolds

Considering the foregoing, there are two different and complex processes: first, scaffold degradation, which decreases its volume and mechanical properties; second, tissue regeneration that increases stiffness and mechanical resistance of new bone. Therefore, experimental [217–225] and computational models are required to show the system evolution over time and to help to identify the optimal initial properties of the scaffold when it is implanted [78, 79, 108, 111, 114, 226–235].

Computer simulations allow analyzing scaffold properties and their effect on growth rate and mechanical behavior of the tissue. Those models vary as different properties, assumptions, domains, and solving approaches are considered. From the geometric point of view the most studied property is porosity [63, 236, 237]. The development of additive manufacturing methods has generated interest in the effect of the shape and size of the scaffold pores. In those studies, a representative volume element (RVE), instead of the whole model of the scaffold, is studied [57, 78]. Simulations can be developed for different processes at different scales. At a nanoscale level, the mechanisms of cell adhesion to the walls can be studied [80, 81]. In the microscale, the effect of the shape and size of the pores can be considered [57, 78], and, at the macroscale, the mechanical behavior of the scaffold [77, 79, 238]. Finally, the use of homogenization and multiscale methods has allowed the researching of various phenomena influencing the process of bone regeneration [111, 239, 240] like substances transport [241, 242]. Some examples of computational mechanobiological models for fracture healing and bone regeneration on porous scaffolds are listed in Table 6.

## 5. Discussion

The global bone graft substitutes market is actually growing, mainly due to the population needs and the improvement of the health services. In this context, design and manufacture of biodegradable scaffolds are one of the major research and development interests in tissue engineering. This paper gives a review about the scaffold design considerations and requirements, the biomaterials that can be selected for a biodegradable scaffold and their related manufacturing processes.

During the scaffold design process there are many considerations to be made: biofunctionality, biocompatibility, biodegradability, mechanical properties, and porosity are among the most important ones. Designing a biodegradable scaffold is a complex process in three ways. First, there are contradictions between the design parameters which must be solved, for example, high porosity versus high mechanical stiffness. Second, the scaffold must be designed using patient-specific parameter values in order to satisfy its functional requirements; thus it is necessary to estimate individual porosity, pore size, and mechanical properties of the affected tissue. Third, the scaffold has to be designed as easy as possible to manufacture; therefore design for manufacturability concepts must be taken into account.

This review discussed many biomaterials and their manufacturing processes for biodegradable scaffold fabrication, but limited work has been done in order to obtain biomaterials with patient-specific degradation rate. One of the future challenges in bone tissue engineering is to design and to manufacture biodegradable scaffolds with a homogeneous growth rate over their entire volume, using pore size gradients or specific distributions of embedded growth factors. This requires manufacturing processes with higher resolution and biofabrication capabilities.

The mechanobiological computational models of the bone regeneration and remodeling processes can assist the design of biodegradable scaffolds because they can help to understand the effect of scaffold properties on bone ingrowth; therefore, their results can be used to optimize the scaffold structure in order to meet patient-specific mechanical and pore characteristics. A disadvantage of these models is that they involve many parameters whose values have to be estimated with* in vitro* or* in vivo* experimentation. It is necessary to rationalize the number of model parameters without loss of reliability of the numerical results.

## Figures and Tables

**Table 1:** Mechanical properties of bone. From Bandyopadyay-Ghosh [39] and Knudson [40].

Property	Cortical bone	Cancellous bone
Tensile strength (MPa)	50–150	10–100
Compressive strength (MPa)	130–230	2–12
Young's modulus (GPa)	7–30	0.02–0.5
Strain to failure (%)	1–3	5–7
Shear strength (MPa)	53–70	
Shear modulus (GPa)	3	

**Table 2:** Mechanical properties of typical polymers and copolymers for tissue engineering. From Maurus and Kaeding, Wu et al., and Middleton and Tipton [131, 137, 141].

Materials	Compressive/tensile strength (MPa)	Young's modulus (GPa)	Elongation (%)	Melting point (°C)	Glass-transition temp (°C)	Loss of strength (months)	Loss of mass (months)
PLLA poly(L-lactide)	28–2300	4.8	5–10	175	60–65	6	24–68

PDLLA poly(DL-lactide)	29–150	1.9	3–10	165–180	40–69	1-2	12–16

PGA poly(glycolide)	350–920	12.5	15–20	200	35–40	1-2	6–12

85/15 DLPLG poly(DL-lactide-co-glycolide)					50–55		5-6
75/25 DLPLG poly(DL-lactide-co-glycolide)	41.4–55.2	2.0	3–10	Amorphous	50–55	1-2	4-5
65/35 DLPLG poly(DL-lactide-co-glycolide)					45–50		3-4
50/50 DLPLG poly(DL-lactide-co-glycolide)					45–50		1-2

PCL poly(*ε*-caprolactone)	23	0.4	300–500	57	50–60	9–12	>24

**Table 3:** Porous biocomposites used for bone tissue engineering. From Chen et al. [142] and Wahl and Czernuszka [143].

Biocomposite		Percentage of ceramic (wt.%)	Porosity (%)	Pore size (*μ*m)	Strength (MPa)	Modulus (MPa)	Ultimate strain (%)
Amorphous CaP	PLGA	28 to 75	75	>100		65	

*β*-TCP	Chitosan-gelatin	10 to 70		322 to 355	0.32 to 0.88	3.94 to 10.88	

HA	PLLA	50	85 to 96	100 × 300	0.39	10 to 14	
	PLGA	60 to 75	81 to 91	800 to 1800	0.07 to 0.22	2 to 7.5	
	PLGA		30 to 40	110 to 150		337 to 1459	
	Collagen	Variable	~0	~0	34–60	0.44–2.82	

	PLG	75	43	89	0.42	51	
Bioglass	PLLA	20 to 50	77 to 80	Approximately 100 (macro); approximately 10 (micro)	1.5 to 3.9	137 to 260	1.1 to 13.7
	PLG	0.1 to 1		50 to 300			
	PDLLA	5 to 29	94	Approximately 100 (macro); 10 to 50 (micro)	0.07 to 0.08	0.65 to 1.2	7.21 to 13.3

Phosphate glass A/W	PLA-PDLLA	40	93 to 97	98 to 154	0.017 to 0.020	0.075 to 0.12	
	PDLLA	20 to 40	85.5 to 95.2				

Bioglass	PGS	90	>90	300 to 500	0.4 to 1.0		

**Table 4:** Properties of bone graft substitutes. Adapted from Ma and Elisseeff [150] and Brown et al. [151].

Property	Allograft	Polymers	Ceramics	Composites	Cell based therapies	Growth factors
Biocompatibility	Yes	Yes	Yes	Yes	Yes	Yes
Osteoconductivity	Yes	Yes	Yes	Yes	No	No
Osteoinductivity	Yes	No	No	Yes	No	Yes
Osteogenicity	Yes	No	No	No	Yes	No
Osteointegrity	Yes	No	Yes	Yes	Yes	No
Mechanical match	No	Yes	Yes	Yes	No	No

**Table 5:** Resorption mechanisms for biomaterials for scaffolds used in bone regeneration. From Bohner [37]^*^.

Material type	Material	Degradation mechanism
	Bioglass	Generally: very limited degradation through partial dissolution
	Plaster of Paris (= calcium sulphate hemihydrate, CSH) Gypsum	Dissolution
Ceramic	Dicalcium phosphate dehydrate (= calcium sulphate dihydrate, CSD)	Dissolution and/or conversion into an apatite
	Calcium carbonate	Dissolution or cell-mediated depending on the mineral phase
	Dicalcium phosphate (DCP) Octacalcium phosphate (OCP) *β*-Tricalcium phosphate (*β*-TCP) Biphasic calcium phosphate (BCP) Precipitated hydroxyapatite crystals *β*-Calcium pyrophosphate (*β*-CPP; *β*-Ca2P2O7)	Cell-mediated
	Sintered hydroxyapatite	Practically no degradation

Metal	Magnesium (alloy)	Corrosion
	Iron (alloy)	Corrosion
	Tantalum, titanium	Practically no degradation

Polymer	Polylactides, polyglycolides Polycaprolactone	Hydrolysis
	Cellulose Hyaluronan Fibrin Collagen Chitosan	Transport to lymph nodes Hyaluronidase Plasmin Collagenase Lysozyme

^∗^Reprinted from Materials Today, with permission from Elsevier [37].

**Table 6:** Computational mechanobiological models for fracture healing and bone regeneration on scaffolds.

Modeled phenomena	Input variable	Output variables	Material	Cells considered	Reference
Fluid motion of a bone substitute applied to the high tibial osteotomy with three different wedge sizes	Fluid-induced shear stress	Elastic modulus, Poisson's ratio, porosity, and permeability values that optimize the internal fluid motion	Not specified	Not specified	[152]

Cell growth *In vitro* versus *in silico *	Local oxygen tension	Cell density	PLGA	Preosteoblast	[153]

Cell differentiation and proliferation on biodegradable scaffold	Shear strain and fluidic velocity	Cell differentiation Cell growth Mechanical properties	PLGA	Mesenchymal cells Osteoblast Osteoclast Chondrocyte Fibroblast	[58]

Cell growth on porous scaffolds	Cell density	Cell density Pressure	Not specified	Not specified	[154]

Cell growth and distribution	Cell density	Cell density and distribution	Not specified	Not specified	[155]

Cell differentiation and proliferation on biodegradable scaffold	Porosity, Young's modulus, and dissolution rate Shear strain and fluidic velocity	Cell differentiation	PLGA	Mesenchymal cells Osteoblast Osteoclast Chondrocyte Fibroblast	[63]

Cell differentiation and proliferation on biodegradable scaffold	Scaffold stiffness, porosity, resorption kinetics, pore size, and preseeding	Cell growth Scaffold mass loss Permeability Porosity	Polymer	Not specified	[156]

Mechanical behavior and drug delivery	Stress loads according to different position *invivo *	Drug release Stress	Hydroxyapatite	Not specified	[157]

Cell growth and differentiation over implant porous surface	Force	Cell differentiation	Not specified	Mesenchymal cells Osteoblast Osteoclast Chondrocyte Fibroblast	[158]

Proliferation and hypertrophy of chondrocytes in the growth plate	Stress	Cell proliferation	Not specified	Chondrocyte	[159]
